# First records of *Amblyomma geayi* and *Amblyomma varium* (Ixodida: Ixodidae) parasitizing sloths in Nicaragua

**DOI:** 10.1007/s10493-025-01032-8

**Published:** 2025-05-28

**Authors:** Juan J. Oporta-López, Carlos Eduardo Molina Vargas, Carmen Guzmán-Cornejo, Adriana Troyo, Filipe Dantas-Torres

**Affiliations:** 1 Independent researcher, Juigalpa, Chontales, Nicaragua; 2 Zoológico Thomas Belt, Juigalpa, Chontales, Nicaragua; 3 Laboratorio de Acarología, Departamento de Biología Comparada, Facultad de Ciencias, Universidad Nacional Autónoma de México, Ciudad de México, México; 4 Laboratorio de Investigación en Vectores, Centro de Investigación en Enfermedades Tropicales, Facultad de Microbiología, Universidad de Costa Rica (UCR), San Pedro de Montes de Oca, San José, Costa Rica; 5 Laboratory of Immunoparasitology, Department of Immunology, Aggeu Magalhães Institute, Oswaldo Cruz Foundation (Fiocruz), Recife, PE Brazil

**Keywords:** Ticks, Wildlife, Mammals, *Bradypus*, *Choloepus*, Central America

## Abstract

Ticks of the genus *Amblyomma* parasitize a wide variety of terrestrial vertebrate hosts, including humans. In Nicaragua, 14 species of ticks of the genus *Amblyomma* have been recorded. To our knowledge, no published records of ticks on sloths exist. However, *A. varium*, which parasitizes these hosts, was reported without an associated host. During 2023 and 2024, five sloths of two species (*Bradypus variegatus* and *Choloepus hoffmanni*) were rescued from four localities belonging to three Departments of Nicaragua. Two species of ticks were collected from the sloths, *Amblyomma geayi* and *Amblyomma varium*. Both records of ticks represent the first for Nicaragua in association with sloths. In this way, the richness of ticks of the genus *Amblyomma* in Nicaragua increases to 15 species.

## Introduction

The genus *Amblyomma* (Ixodida: Ixodidae) currently includes approximately 138 species, with 68 occurring in the Neotropical region and 55 exclusive to this region (Guglielmone et al. 2023; Soares et al. 2023; Ali et al. 2024; Kwak et al. 2025; Robbins et al. 2025).

Ticks of the genus *Amblyomma* parasitize a great diversity of terrestrial vertebrate hosts, including humans (Guglielmone et al. 2014). They are also relevant to animal and human health due to their role as vectors of pathogens, such as viruses, bacteria, and protozoa (de la Fuente et al. 2008; Dantas-Torres et al. 2012).

In Nicaragua, 14 species of ticks of the genus *Amblyomma* have been recorded: *Amblyomma auricularium* (Conil, 1878), *Amblyomma coelebs* Neumann, 1899, *Amblyomma dissimile* Koch, 1844, *Amblyomma maculatum* Koch, 1844, *Amblyomma mixtum* Koch, 1844, *Amblyomma nodosum* Neumann, 1899, *Amblyomma oblongoguttatum* Koch, 1944, *Amblyomma ovale* Koch, 1844, *Amblyomma parvum* Aragão, 1908, *Amblyomma sabanerae* Stoll, 1894, *Amblyomma scutatum* Neumann, 1899, *Amblyomma tapirellum* Dunn, 1933, *Amblyomma tenellum* Koch, 1844, and *Amblyomma varium* (Guglielmone et al. 2023). These represent 10.14% of the known *Amblyomma* spp. To the best of our knowledge, no records of ticks on sloths in Nicaragua have been published in the scientific literature. Although the giant sloth tick *A. varium* was reported long ago in Panama (Robinson 1926), there was no information on the host.

In Central and South America, about ten species of ticks of the genus *Amblyomma* have been reported parasitizing different species of sloths (Luz et al. 2020; Nava et al. 2017; Urushiyama et al. 2020; Bernardes et al. 2022). Particularly in Nicaragua, two species of sloths of two different families are present: the three-toed brown-throated sloth *Bradypus variegatus* (Pilosa: Bradypodidae) and Hoffmann´s *Choloepus hoffmanni* (Pilosa: Choloepodidae) (McCarthy et al. 1999). The International Union for Conservation of Nature (IUNC) Red List of Threatened Species considers both sloth species to be of least concern (Moraes-Barros et al. 2022; Please et al. 2022). Based on the above, the objective of this study was to identify and report the species of ticks collected from five sloths rescued in different geographic regions of Nicaragua.

## Materials and methods

**Fig. 1:**
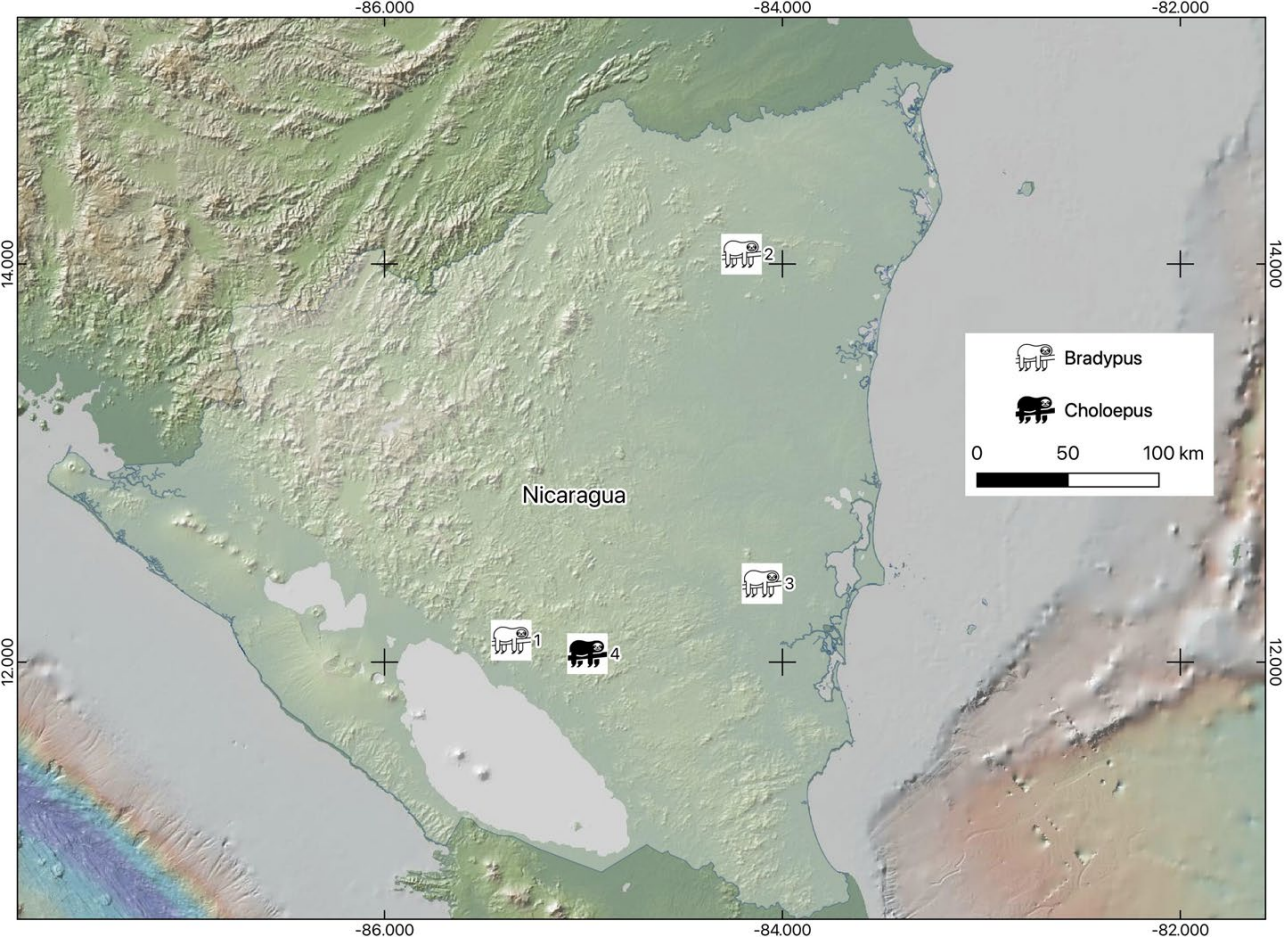
Ticks collected from sloths rescued from four localities belonging to three Departments of Nicaragua: (1) Juigalpa, Chontales, (2) Riscos de Oro, Rosita, (3) Monte Rosa, El Rama, (4) Villa Sandino, Chontales

During June 2023, personnel of the Thomas Belt Zoo in Juigalpa city, Chontales department, Nicaragua, rescued three sloths for relocation purposes: a male *B. variegatus* from the community of Riscos de Oro, Rosita municipality of the Región Autónoma de la Costa Caribe Norte, and two *C. hoffmanni* (a female with her brood) from the municipality of Villa Sandino, Chontales department. In June 2024, a female *B. variegatus* was captured on a farm in Monte Rosa, El Rama municipality of the Región Autónoma de la Costa Caribe Sur; later in the same month, a male sloth of the same species was recovered while being offered for sale on the streets of Juigalpa (Fig. 1).

The mammals were physically contained, and both ventral and dorsal surfaces of the body were inspected for ticks. The ticks observed were collected using fine-tipped forceps and conserved in 95% ethanol at room temperature. Later, the ticks were observed using a stereomicroscope (LW Scientific Microscope Z4 Zoom System). Morphological characteristics of the ticks (nymphs and adults) were used for their identification with the aid of taxonomic keys (Martins et al. 2010; Guzmán-Cornejo et al. 2011; Bermúdez et al. 2018; Dantas-Torres et al. 2019). Once a species was reached, the specimens were compared with species descriptions (Castilho-Onofrio et al. 2008; Martins et al. 2010, 2013).

The ticks were deposited in the collection of invertebrates of medical-veterinary interest (accession number A-11) of the Biology Laboratory of the Universidad Nacional Agraria, Centro regional Juigalpa, Nicaragua.

## Results

Nine ticks were collected from the five captured sloths: one male and six nymphs of *A. geayi* from the *B. variegatus* from Riscos de Oro, and two males of *A. varium*, one from the *B. variegatus* from Juigalpa and the other from the *C. hoffmanni* female from Villa Sandino. The sloth captured in Monte Rosa and the brood from Villa Sandino were negative for ticks.

**Fig. 2:**
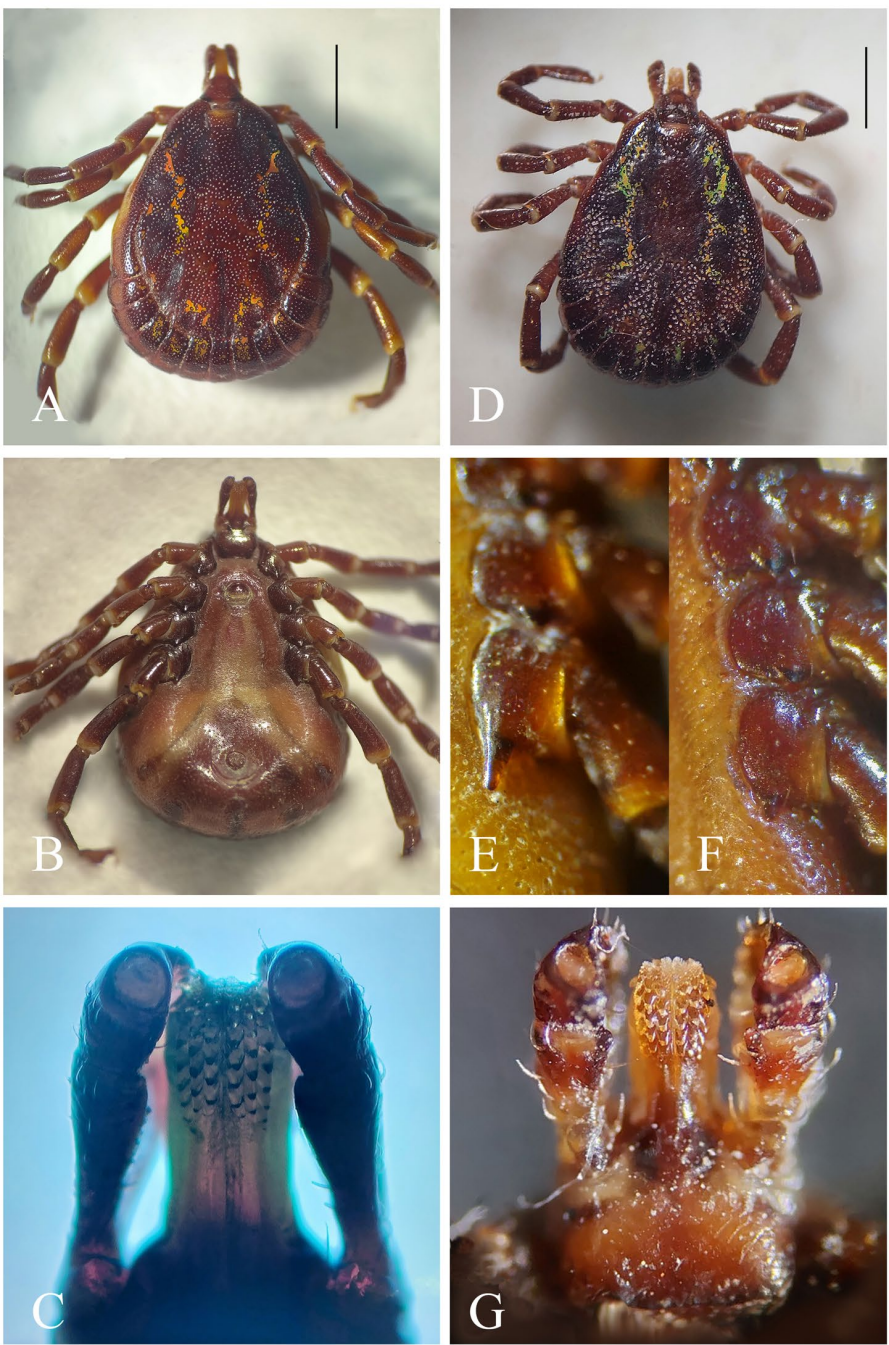
*Amblyomma geayi*, male: (**A**) Dorsal view, marginal groove complete; bar length = 2150 μm. (**B**) Ventral view, five plates on the ventral back. (**C**) Hypostomal dentition 3/3. *Amblyomma varium*, male: (**D**) Dorsal view, marginal groove incomplete; bar length = 1700 μm. (**E**) Long spur on coxa IV (from *C. hoffmanni*). (**F**) Short spur on coxa IV (from *B. variegatus*). (**G**) Hypostomal dentition 3/3

The characters observed in the male of *A. geayi* corresponded to those reported by Labruna et al. (2009): large oval body, narrowing anteriorly; marginal groove complete, starting anterior to mid-length of body; festoons large and well defined, and ornate; dorsal scutum ornamented with irregular yellow-green spots on the sides, punctations deep and evenly distributed; basis capituli triangular, without cornua; hypostome spatulated with dentition 3/3; genital opening at the level of coxae II; ventral plates large; coxae I with two short, flat, and sub-equal spurs, the external longer than internal; coxae II-IV with one triangular spur, longest on coxa IV (Figs. 2A-C).

**Fig. 3:**
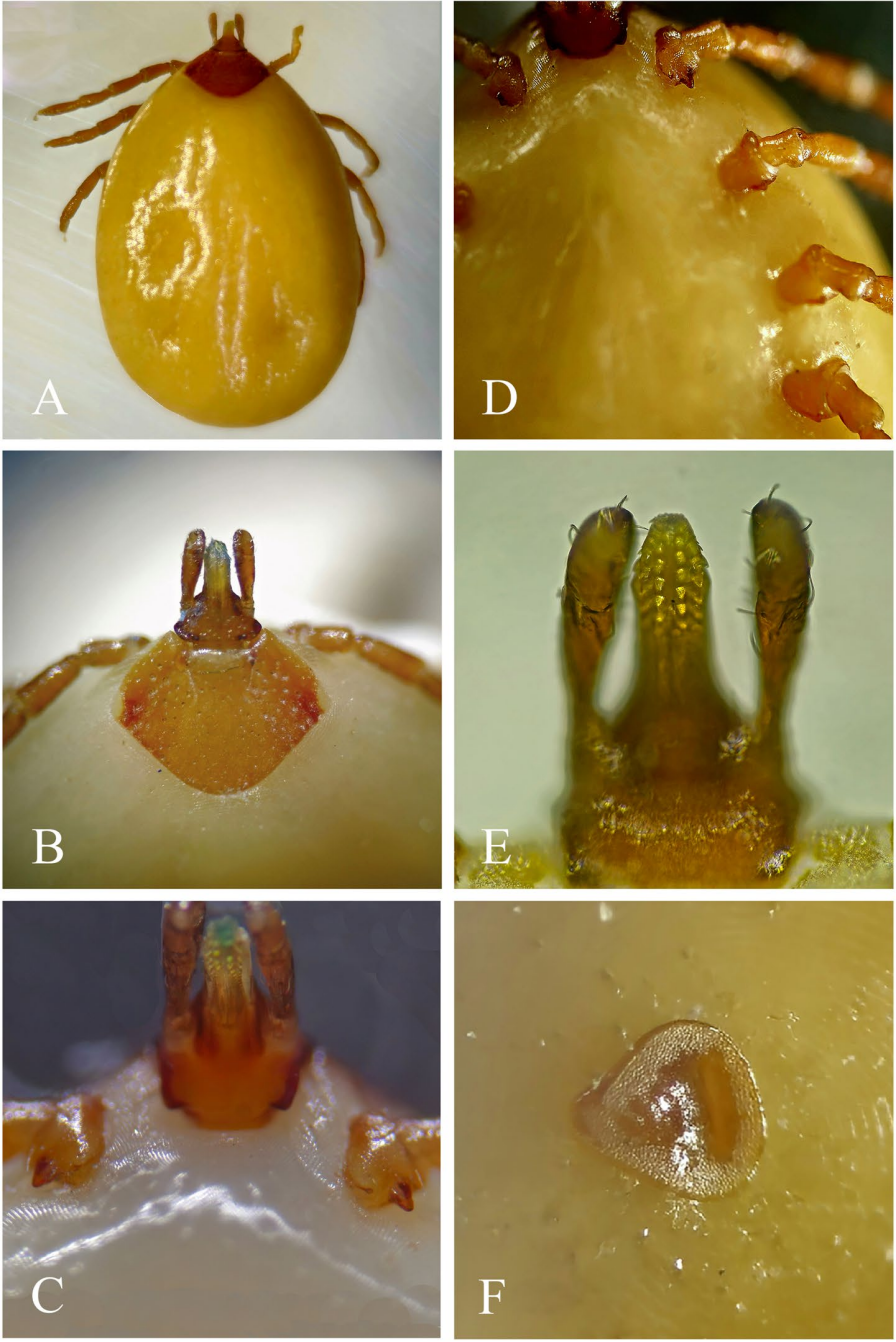
*Amblyomma geayi*, nymph. (**A**) Dorsal view. (**B**) Scutum with numerous punctations. (**C**) Coxa I with two short spurs, the internal almost vestigial. (**D**) Coxae II-IV with a small triangular spur. (**E**) Hypostomal dentition 2/2. (**F**) Spiracular plate

All collected nymphs (*n* = 6) were engorged and were identified as *A. geayi* due to their morphological correspondence with the characters indicated by Martins et al. (2013), such as: scutum [length 0.9–1.2 mm (mean 1.08); width 1.2 mm (1.2)] without ornamentation and with numerous punctations; non-orbited eyes in lateral scutal angles at the level of the average scutal length; spiracular plate with triangular shape with rounded angles, with a discrete dorsal prolongation; base of dorsal capitulum subtriangular [length: 0.3–0.4 mm (0.33); width 0.3–0.4 mm (0.37)], with a straight posterior margin, without horns, ventrally convex posterior margin, auricles as small rounded posterolateral projections; hypostome rounded apically [length 0.28–0.32 mm (0.3)], dentition 2/2; coxa I with two short spurs, the external wider than the internal, the internal almost vestigial; coxae II-IV with a small triangular spur (Figs. 3A-F).

In turn, the morphological features observed in males of *A. varium* were similar to those reported by Castilho-Onofrio et al. (2008), Martins et al. (2013), and Bermúdez et al. (2018), such as: large oval body with marginal groove absent; festoons large and defined; dorsal scutum ornamented with irregular yellow-green spots on the sides; punctations numerous, absent in median region, and deeper in lateral margins; dorsal base of capitulum rectangular with short cornua; hypostome spatulated with dentition 3/3; genital opening at level of coxae II; coxa I with two short and sub-equal spurs, coxae II-IV with one rounded spur; spur of coxa IV varying in length (Figs. 2D-G).

The *C. hoffmanni* female harbored a male of *A. varium* with a long spur on coxa IV (Fig. 2E), while the one found on the *B. variegatus* male presented a short spur on coxa IV (Fig. 2F).

## Discussion

The richness of ticks of the genus *Amblyomma* in Nicaragua before this study was 14 species, including *A. varium*, which was recorded in the country under the name *Amblyomma crassipunctatum* Stoll, 1890; a species that was referred to without further information about its host, and that is currently considered a synonym of *A. varium* (Robinson 1926; Camicas et al. 1998; Guglielmone and Nava 2014).

*Amblyomma geayi* is a Neotropical species, and its distribution before the present report included South America (Brazil, Colombia, French Guiana, Guyana, Peru, Suriname, Venezuela) and the southern portion of Central America (Costa Rica and Panama) (Souza et al. 2016; Guglielmone et al. 2023). In addition, there is a record from this tick species on a Linnaeus´s two-toed sloth, *C. didactylus*, imported to Japan (Urushiyama et al. 2020). The current report of *A. geayi* in Nicaragua increases the tick richness in the country to 15 species and represents the northernmost report for the species (Guglielmone et al. 2023).

*Amblyomma varium* parasitizes almost exclusively species of Bradypodidae and Choloepodidae (= Megalonychidae) (Castilho-Onofrio et al. 2008). This species has occasionally been found on other hosts, but these records are considered accidental (Dantas-Torres et al. 2021). *Amblyomma varium* is also a Neotropical tick found in South and Central America (Brazil, Colombia, Costa Rica, Ecuador, French Guiana, Guatemala, Guyana, Nicaragua, Panama, Peru, and Venezuela) (Guglielmone et al. 2023). It should be noted that the male of *A. varium* with the long spur on coxa IV was found on *B. variegatus*, while the male with the short spur on coxa IV was found on *C. hoffmanni*; this variation was considered an intraspecific polymorphic character for this tick species. Castilho-Onofrio et al. (2008) found that specimens with long and short spurs were genetically identical based on 12 S rDNA gene sequences.

*Amblyomma geayi*, *Amblyomma longirostre* Koch, 1844, and *Amblyomma parkeri* Fonseca and Aragão, 1952, are phylogenetically close species that are often confused morphologically. Although they share morphological characteristics, the male of *A. geayi* can be separated from *A. longirostre* by its complete marginal groove, which is incomplete in *A. longirostre*.

As for differences with *A. parkeri*, the scutum in *A. geayi* is larger (˃5.0 mm) than that of *A. parkeri* (˂5.0 mm); in addition, the marginal groove in *A. geayi* forms an obtuse angle at the level of the spiracular plate that gives the shield a diamond appearance, whereas a rounded angle is formed at the level of the spiracular plate in *A. parkeri*, giving it an oval shape (Labruna et al. 2009). In addition to morphological differences, *A. parkeri* and *A. longirostre* mainly parasitize porcupines, whereas *A. geayi* has Bradypodidae species as its main hosts (Labruna et al. 2009; Guglielmone and Robbins 2018).

Aside from Bradypodidae, vertebrate hosts for *A. geayi* also include species of Erethizontidae and Choloepodidae (Guglielmone and Robbins 2018). Other reports mention Didelphidae, Trochilidae, and several families of Passeriformes (Guglielmone and Robbins 2018). On the other hand, adults of *A. varium* have been recorded mainly in Bradypodidae and Choloepodidae, and nymphs have also been collected from Bradypodidae. Other hosts of larval, nymphal, and adult stages include: Didelphidae, Tayassuidae, Procyonidae, Caviidae, Iguanidae, Pipridae, Echimyidae, Icteridae, Bucconidae, and several families of Passeriformes (Guglielmone and Robbins 2018).

It has been speculated that *A. geayi* could have zoonotic importance as a potential vector of *Coxiella burnetii* (Davoust et al. 2014), but it remains to be proven. Indeed, although there are some records of *A. geayi* nymphs parasitizing humans, these records also need confirmation (Guglielmone et al. 2014; Guglielmone and Robbins 2018). In addition, *A. geayi* parasitizing other vertebrates can harbor *Rickettsia amblyommatis*, although the pathogenic potential of this bacterium remains poorly understood (Dolz et al. 2019; Moreira-Soto et al. 2023).

Similarly, *A. varium* is considered a very rare parasite of humans, and some records of nymphs parasitizing humans need confirmation (Guglielmone et al. 2014; Guglielmone and Robbins 2018). While *R. rickettsii* was molecularly detected in a nymph of *A. varium* found parasitizing a human in Costa Rica (Troyo et al. 2016), this tick is not considered involved in transmitting spotted fever to humans.

In conclusion, this report represents the first description of ticks in Nicaraguan sloths, increases the number of tick species in this country, and extends the northernmost limit of *A. geayi*.

## Data Availability

We declare all data is being provided within this manuscript.
